# How Reliable Is Pyramidal Wavefront-Based Sensor Aberrometry in Measuring the In Vivo Optical Behaviour of Multifocal IOLs?

**DOI:** 10.3390/s23073534

**Published:** 2023-03-28

**Authors:** Francesco D’Oria, Giacomo Scotti, Alessandra Sborgia, Francesco Boscia, Giovanni Alessio

**Affiliations:** Section of Ophthalmology, Department of Basic Medical Science, Neuroscience and Sense Organs, University of Bari, 70121 Bari, Italy

**Keywords:** cataract surgery, pyramidal aberrometry, multifocal IOL, extended depth-of-focus IOL

## Abstract

Cataract or refractive lens surgery, along with the implantation of multifocal intraocular lenses (MF-IOL), enables a complete range of functional far, near and intermediate vision. Refractive, diffractive and extended depth of focus (EDoF) or combination of these principles represent the technology used to obtain this multifocality. Aberrometry makes it possible to study the aberrations induced by MF-IOLs. Among the different optical principles available to measure ocular aberrations, pyramidal wavefront-based sensor (PWS) aberrometry shows the highest resolution with MF-IOLs. Retinal image quality measured by a PWS aberrometer differed significantly according to the technology of the implanted lens. Monofocal and diffractive lenses showed the highest values of far-distance retinal image quality, followed by refractive and EDoF lenses; however, retinal image quality analysed in diffractive lenses appears to be more dependent on residual refractive error. Considering this limitation, PWS-aberrometry could be used to compare diffractive lenses. Nevertheless, further studies are needed to provide additional information about the clinical retinal image quality of MF-IOLs and to help surgeons in the important preoperative selection of IOLs.

## 1. Introduction

Cataract surgery, initially adopted for the sole purpose of treating disease, has, over the years, developed ambitious visual and refractive objectives. Nowadays, this surgical technique, along with intraocular lens (IOL) implantation, has been used not only for the purpose of resolving cataract disease with visual rehabilitation but also to improve patients’ quality of life, allowing them to perform all daily activities without the need for glasses [1,2,3]. A second milestone was the development of multifocal IOLs (MF-IOLs) to overcome pseudophakic presbyopia [4]. MF-IOLs, using different optical designs, split light into multiple foci, simultaneously focusing the retina on images placed at different distances, thereby obtaining a complete range of vision at far, intermediate and near distances. MF-IOL optics can be based on a refractive, diffractive, or extended depth-of-focus (EDoF) technology or combination thereof.

**Figure 1:**
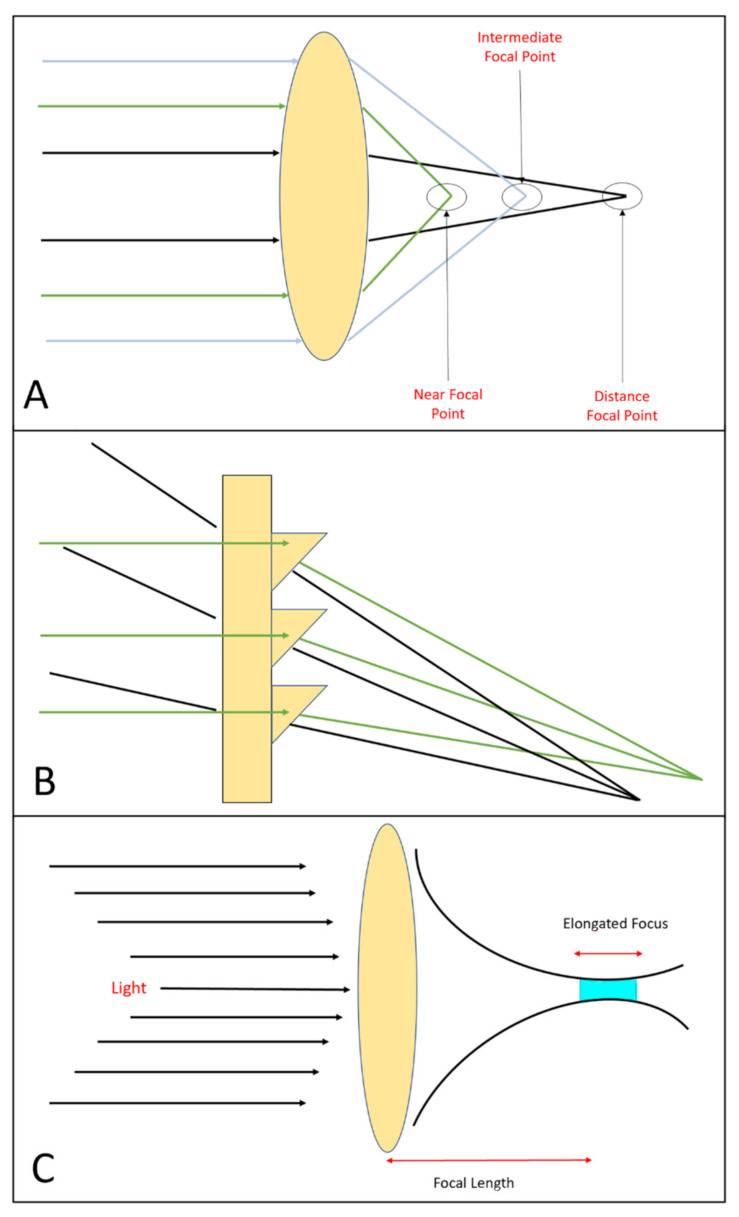
(**A**) Refractive lens design with three zones that concentrates light rays from the intermediate distance (light blue arrows), near distance (green arrows) and far distance (black arrows). (**B**) Image showing the principle of a diffractive lens. Light travels slower on the side of the step of the lens compared to the speed of light moving through the aqueous area, resulting in two foci: one for near vision and one for far vision. (**C**) Extended depth-of-focus (EDoF) lens design, which works by creating a single elongated focal point rather than several focal points to enhance the depth of focus.

Refractive lenses obtain multifocality using the optical principle of refraction; their optics are determined by areas that differ in terms of refractive power (Figure 1A). These types of multifocal lenses can be rotationally symmetric or rotationally asymmetric. Rotationally symmetric MF-IOLs have a structure with concentric rings, and each ring, thanks to its refractive power, constitutes a different focus zone [5]. On the other hand, rotationally asymmetric MF-IOLs have a lower segment which, thanks to its refractive power, provides near vision by a calculated magnitude of intraocular coma [6].

In diffractive multifocal lenses, the effect of the diffraction of light is used. As first described by Fresnel in 1822, when the wavefront meets an obstacle, it is altered in phase and amplitude, creating a diffractive pattern [4]. Thus, by placing diffractive microstructures in concentric zones and reducing the distance between these zones from the centre to the periphery, two or three main foci are determined simultaneously (Figure 1B).

A more recent technology involves the use of EDoF IOLs. The optical principle is to create a single elongated focal point to enhance the depth of focus, in contrast to monofocal IOLs (in which light is focused on one single point, i.e., the far distance) or MF-IOLs (with two or three discrete points for near, intermediate and distance vision).These lenses, by creating a single elongated focal point (Figure 1C), attempt to overcome the limitation of dividing the light into multiple foci, as well as the problems of secondary glare and halos; instead, they increase the depth of focus [7].

## 2. Measurement of Aberrations

The eye is an optical system that focus light rays onto the retina to create images using several optical elements. Imperfections in the components and materials of these elements may cause light rays to deviate from the desired path. These deviations, known as optical aberrations, decrease the visual performance of the optical system. Cornea and crystalline lens are the most important sources of aberrations, but IOLs also cause light rays to deviate from their path [8]. Night vision halos and glare are typical visual disturbances of highly aberrated eyes. Ocular aberrations are one of the principal causes of retinal image degradation. Due to their impact on the patient’s vision, it is important to measure ocular aberrations with high precision in clinical practice [8,9].

Aberrations are quantified using Zernike and Fourier expansion series polynomials [10]. Zernike polynomials are used to represent and classify ocular aberrations. Since they are schematically disposed to create a pyramid, we distinguish low-order aberrations (LOA, at the edge of the pyramid) and high-order aberrations (HOA, going to the base). Defocus and astigmatism (top of the pyramid) are the simplest and the most common ocular aberrations that can be detected and quantified using an autorefractometer or a skiascopy. Although these aberrations can deeply affect vision quality of patients, they are the only ones corrected using spectacles, contact lenses or refractive surgery. Zernike polynomials cannot describe the impact of each single aberration on visual function or provide a precise description of the eye’s image-forming properties. To overcome this limitation, the point spread function (PSF) and the Strehl ratio (SR) are used to describe the retinal quality of images and the optical performance of the eye. PSF describes how a point of light is projected into the retina, indicating the dimness of the retinal image. SR is the ratio of the peak height of the PSF to the peak height for the same optical system if it were diffraction-limited [11]. Different optical principles can be used, such as the Hartmann–Shack (H-S) method, the Tscherning principle, ray tracing and pyramidal wavefront-based sensor (PWS) aberrometry.

### 2.1. Hartmann–Shack Method

The H-S method is a parallel, double-pass method using backward projections of a narrow laser beam sent into the eye following the visual axis that reflects on the retina. This reflection illuminates the pupil area from behind. The outgoing light is then guided through a set of relay lenses that projects the pupil plane onto an array of tiny lenses that splits up the wavefront into several individually focused spots on a charged coupled device camera. The wavefront slopes are determined by analysing the spot displacement due to the focal shift [12]. Muñoz et al. [13] used an H-S aberrometer to evaluate the optical quality of 174 eyes implanted with ReZoom (AMO, Abbott Park, IL, USA) multifocal IOLs after cataract surgery. Its multifocality consists of five concentric refractive areas alternating distance-dominant (zones 1, 3 and 5) and near-dominant (zones 2 and 4), with a near power of +3.5 D. Aberrometric analysis indicated the poor optical quality of these IOLs. However, optical quality measures obtained using an H-S aberrometer in eyes implanted with MF-IOLs must be interpreted with care. In fact, the transition between different refractive zones creates distortion of the spots, leading to erratic measurements. As described by Charman et al. [14], this seems to be particularly important for diffractive MF-IOLs but also present in refractive MF-IOLs.

### 2.2. Tscherning Principle

The Tscherning principle is a parallel, double-pass method using forward projection. In this case, a wide laser beam passes through a screen with many round holes, generating a group of laser beams that enter the eye. A system of lenses allows enables analysis of the retinal spot pattern for wavefront analysis. These types of aberrometers provide reproducible measurements in normal eyes but are limited in eyes with significant amounts of aberrations due to overlapping of the spots [15]. Cade et al. [16] compared the agreement among four commonly used aberrometers: the Wavelight Allegro analyser (Wavelight, Erlangen, Germany), which is based on the Tscherning principle; the Alcon LADARWave (Alcon, Fort Worth, TX, USA); the Visx WaveScan (VISX, Santa Clara, CA, USA) and the Zywave (Baush & Lomb, Rochester, NY, USA), which is based on the above mentioned H–S method. The analysis of the data showed that there was good agreement between LOA and HOA for all four wavefront analysers. However, statistically significant differences were measured between the aberrometers regarding total HOA (*p* = 0.001), spherical aberration (SA) (*p* = 0.001) and horizontal coma (*p* = 0.001).

### 2.3. Ray Tracing

The ray-tracing principle is a serial, double-pass method using forward projections whereby a narrow laser beam is directed into the eye parallel to the line of sight by means of an x–y scanner. The x–y scanner moves the beam to cover the whole pupil area. Ocular aberrations cause a focal shift of the retinal image acquired on a linear array of photodetectors [17]. The direction that the light beams taken when entering and leaving allows for a reconstruction of the real wavefront error. This technology is less influenced by the multifocal design of the IOL because of its sequential shooting, as described by Jun et al. [18], who evaluated the optical quality of 87 eyes of 87 patients using the iTrace technology (Tracey Technology, Houston, TX, USA) one of a ray-tracing aberrometer. Patients were implanted with monofocal spherical IOLs (SN60AT; Alcon, Inc.; group 1), monofocal aspheric IOLs (SN60WF; Alcon, Inc.; group 2) and multifocal aspheric IOLs (Acrysof ReSTOR, SN6AD1; Alcon, Inc.; group 3). Results showed that the SA of patients implanted with spherical monofocal IOLs had a positive value proportional to the IOL diopter, while monofocal aspheric and multifocal aspheric IOLs had negative SA values similar to those designated by the manufacturer for pupil sizes of 5 and 6 mm.

### 2.4. Pyramidal Wavefront-Based Sensor Aberrometry

A new generation of aberrometers was created to evaluate ocular aberrations using a PWS technology based on the Foucault knife-edge test (Osiris, Costruzione Strumenti Oftalmici (CSO), Firenze, Italy). In a pyramidal aberrometry system, a four-faced PWS provides the wavefront gradients in two orthogonal directions, and four sub-pupils are distributed according to their intensity. Each sub-pupil performs a Foucault knife-edge test to derive the slope and shape of the wavefront. In contrast to other methods, the wavefront is sampled in the very last stage of the optical path. An H-S aberrometer discretizes the wavefront during the lenslet stage, so the number of measured samples depends on the number of lenses on the lenslet. An H-S sensor generally has 1000–2000 lenses with a resolution of 250–1250 μm; in contrast, the Osiris is sampled with 45,000 points at maximum pupil dilation, which corresponds to a resolution of 41 μm [19].

## 3. Multifocal IOLs Measured by PWS

There are some drawbacks related to the use of MF-IOLs. First, light is divided into multiple foci (light energy is not directed onto a single focus as in natural accommodation), so the light that enters the eye undergoes dispersion, and a small percentage of it becomes scattered and lost, resulting in a somewhat decreased quality of vision compared to monofocal IOLs [20]. Aberrometry makes it possible to study the way the lenses split the light according to their optics. Aberrations induced by MF-IOLs have been studied both ex vivo and using artificial eyes. However, the results of ex vivo studies do not always correlate well with visual outcomes in real practice, and some lenses that perform well ex vivo later show disappointing results in real clinical practice. Therefore, in vivo analysis of intraocular optical quality and aberrations represents a more reliable method of approaching postoperative patient-perceived quality of vision and visual complaints, thereby avoiding problems related to neuroadaptation.

PWS was first used by Oliveira et al. [21] in 2018 to study the optical outcomes of FineVision IOL (PhysIOL, Liege, Belgium) after implantation, the first commercially available diffractive trifocal IOL. The FineVision IOL has an anterior aspheric optic with an SA of −0.11µm with a 6.0 mm pupil to partially reduce whole-eye optical SA [22]. As a proof of the ability of PWS to measure ocular aberrations, the mean ocular SA measured with a pyramid wavefront aberrometer was shown to be as low as 0.10 ± 0.12 µm with a 6.0 mm pupil, leaving a majority of patients with residually limited SA, which may provide a slight increase in the depth of field [18]. Moreover, the authors found no statistically significant correlation between wavefront aberrometry parameters and patient satisfaction outcomes, which were measured by the 10-item Near Activity Vision Questionnaire (NAVQ-10).

Alio et al. [23] compared optical image quality following implantation with different premium IOLs. They studied 194 eyes of 120 patients by analysing the PSF Strehl ratio using a PSW aberrometer at pupillary diameters of 3.0 and 4.0 mm. The following lenses were included in the study: 19 Acrysof SA60ATs (monofocal spherical, Alcon Inc., control group), 19 EDoF Miniwells (SIFI, Catania, Italy), 24 multifocal refractive LENTIS Mplus LS-313 MF30s (Oculentis GmbH), 33 multifocal refractive LENTIS Mplus LS313 MF15s (Oculentis GmbH), 17 Akkolens Lumina accommodative intraocular lenses (Akkolens Clinical B.V., Breda, The Netherlands), 31 multifocal diffractive AT LISA Tri 839MPs (Carl Zeiss Meditec AG, Jena, Germany), 20 multifocal refractive Precizon Presbyopics (Ophtec BV, Groningen, The Netherlands), 20 multifocal diffractive PanOptix (Alcon, Fort Worth, TX, USA) and 11 aspheric monofocal Tecnis Eyhance ICB00s (Johnson & Johnson Vision, Santa Ana, CA, USA). To overcome the problem related to the residual refractive error and the corneal geometry itself, the PSF Strehl ratio excluding second-order aberrations (PSFw2) was calculated, and second-order aberration removal was performed by decomposing the wavefront in terms of Zernike. AT LISA Tri is a multifocal lens that corrects near, intermediate and far vision using a diffractive structure that covers the entire anterior optical surface to generate three wavefronts of varying curvature that emerge from the IOL, with the highest values of far distance retinal image quality at both 3.0 and 4.0 mm pupil sizes (0.52 ± 0.14 and 0.31 ± 0.10), followed by SA60AT (0.41 ± 0.11 and 0.28 ± 0.07) and PanOptix (0.4 ± 0.07 and 0.26 ± 0.04). The lowest PSFw2 Strehl ratios were showed by the Miniwell, Mplus MF30 and Precizon Presbyopic lenses. The Mini Well Ready (SIFI, Catania, Italy) is a one-piece EDoF IOL with a double aspherical design. Specifically, SAs are induced in certain areas of the optic to increase the depth of focus [7]. The Lentis LS-313 (Oculentis, Topcon) is a one-piece foldable bifocal hydrophobic acrylic IOL for intermediate and far vision with a sector-shaped addition of +1.5 diopters (MF15) or +3.00 diopters (MF30) [4]. The Precizon Presbyopic (Ophtec BV) is a one-piece hybrid hydrophobic and hydrophilic with continuous transition focus that obtains a smoother transition between distance and near vision by combining different sectors in the optical zone of the IOL [4].

These results were later confirmed by evaluating the PSFw2 of refractive MF-IOLs in a set of four groups of patients. In this study, AcrySof SA60AT had the highest significant PSFw2 Strehl ratio for both the 3 and 4 mm pupil sizes, followed by LENTIS Mplus 1.5 and a near tie between LENTIS MPLUS 3.0 and Precizon Presbyopic [24]. These findings suggest that the use of a higher addition for the rotationally asymmetric IOL limits the optical quality, with a relative effect on retinal image quality. Similar findings were shown by Vargas et al. [25], who conducted a study that evaluated visual outcomes and satisfaction of patients after blended implantation of rotationally asymmetric MF-IOLs. They studied 40 eyes of 20 patients implanted with Mplus LS-313 +3.00 D in the non-dominant eye and Mplus LS-313 +1.5 D in the dominant eye. Twelve months after surgery, using a PWS aberrometer, they showed that there were significantly more HOAs in the +3.00 D eye.

Al-Amri et al. [26] evaluated clinical retinal image quality of a non-diffractive wavefront-shaping EDoF IOL (AcrySof IQ Vivity, Alcon). The investigated lens is a single piece, hydrophobic aspheric posterior chamber IOL with a modified middle 2.2 mm region within the 6 mm optic that includes two smooth-surface transition elements. It uses exclusive wavefront-shaping technology to provide an extended focal length that mostly directs incoming light through the IOL within a certain range. In this study, AT LISA Tri showed the highest PSF without LOA at both pupil sizes, followed by SA60AT and EDoF Vivity IOLs. On the other hand, the AT LISA Tri retinal image quality (PSF with LOA) was the most severely affected by residual refractive errors (dropped to 0.26 ± 0.06 at 3 mm) compared to the monofocal (0.24 ± 0.07 at 3 mm) and Vivity IOL (0.23 ± 0.06 at 3 mm) groups, which remained stable overall.

## 4. Conclusions

Diffractive lenses show the longest-distance retinal image quality, followed by refractive lenses and EDoF lenses. Nevertheless, diffractive lenses were the most severely affected by residual refractive errors compared to monofocal lenses. A non-diffractive wavefront-shaping EDoF IOL is a new type of lens that is less affected by residual refractive errors. An aberrometer is unable to correctly interpret secondary replicas generated by a diffractive lens, and all the cited works only focused on retinal images in for far vision and not on other distances (near and intermediate), for which measurements are less reliable. Bearing in mind this limitation, the outcomes of the eye implanted with diffractive multifocal IOLS are affected by the same type of bias; in this way, IOLs with the same type of multifocal optic (e.g., multifocal diffractive) can be compared. The same considerations can be applied to multifocal refractive IOLs, considering that they are less affected by the light dispersion that affects the diffractive models.

## 5. Future Directions

The novel aberrometer described herein makes it possible to evaluate, for the first time, the real clinical retinal optical quality of the living human eye once implanted with a multifocal lens. This will be an important topic in the future, once further improvements clarify which lenses can reduce the bias induced by light dispersion in intermediate and near vision, ultimately allowing surgeons to compare different lenses when implanted in the human eye to choose the best lens for surgical procedures according to their aberrometric behaviour and retinal clinical images. Further in vivo studies are needed to evaluate all the available multifocal IOLs and extend the number of studied IOLs in each group. This will represent an important tool to guide surgeons in the important decision making process related to the selection of IOLs.

## Data Availability

No new data were created in this review paper.
